# Integrated bioinformatics analysis shows integrin alpha 3 is a prognostic biomarker for pancreatic cancer

**DOI:** 10.1515/med-2022-0606

**Published:** 2022-12-09

**Authors:** Fangfang Hu, Liangtao Zhao, Yang Wang, Hao Ye, Haodong Tang, Jiahua Zhou

**Affiliations:** Department of Hepatobiliary and Pancreatic Surgery, Zhongda Hospital Affiliated to Southeast University, Nanjing, 210009, China; School of Medicine, Southeast University, Nanjing, 210009, China; Department of Hepatobiliary and Pancreatic Surgery, Zhongda Hospital Affiliated to Southeast University, No. 87 Dingjiaqiao, Gulou District, Nanjing, 210009, China

**Keywords:** pancreatic cancer, integrin alpha 3, meta-analysis, PI3K-Akt signaling pathway, extracellular matrix

## Abstract

Integrin subunit alpha 3 (ITGA3) expression correlates with the development and prognosis of human cancers. This study aimed to investigate the association of ITGA3 expression with pancreatic cancer (PCa) prognosis. The ITGA3 gene expression data were extracted from The Cancer Genome Atlas (TCGA) pancreatic adenocarcinoma (PAAD) cohort and 14 Gene Expression Omnibus microarray datasets. The differences in ITGA3 expression levels between tumor and non-tumor tissues were compared using the Mann–Whitney *U* test. Cox regression analysis and meta-analysis were performed to detect the association of ITGA3 expression with PCa prognosis. ITGA3 expression was higher in tumors than in controls. Tumors with advanced grades (3/4) had higher ITGA3 levels compared with early-grade tumors (1/2). The meta-analysis of the TCGA PAAD cohort and seven microarray datasets (GSE28735, GSE62452, GSE79668, GSE71729, GSE57495, GSE78229, and GSE21501) showed that ITGA3 was a prognostic biomarker in PCa (hazard ratio (HR) = 1.38, 95% confidence interval (CI) 1.26–1.51, *p* < 0.00001). Five ITGA3-related genes, including ITGB1 (HR = 1.6), ITGB5 (HR = 1.6), ITGB6 (HR = 1.6), LAMA3 (HR = 2.1), and CD9 (HR = 2.3), correlated with PCa prognosis significantly (*p* < 0.05). Functional enrichment analysis showed that ITGA3 was related to “hsa04151: PI3K-Akt signaling pathway” and “hsa04510: Focal adhesion.” We concluded that high ITGA3 expression was a potential prognostic biomarker in PCa.

## 1 Introduction

Pancreatic cancer (PCa), also known as pancreatic adenocarcinoma (PAAD), is a highly fatal disease. PCa is predicted to be the second leading cause of cancer-related deaths in the next decade [1,2,3]. Also, the global mortality rate of PCa has increased from over 200,000 deaths in 2005 to over 331,000 deaths in 2012 and over 432,000 deaths in 2018 [4,5,6]. The 5 year overall survival rate of PCa is estimated to be approximately 10%, with little change over the past few decades [3,7,8,9].

The identification of diagnostic biomarkers for PCa might promote the detection of early-stage disease, and the identification of prognostic biomarkers might be an option to improve the treatment and prognosis in PCa patients [10,11,12]. Therefore, many research studies have been performed to identify effective biomarkers for early diagnosis and prognosis improvement in PCa [10,11,12,13,14,15]. The integrin subunit alpha 3 (ITGA3), a member of the integrin family, has been identified as an important prognostic biomarker in several types of human cancers, including PCa [16], breast cancer [17], non-small cell lung cancer [18], and bladder cancer [19]. ITGA3 is an integrin and extracellular matrix (ECM) receptor located on the cell membrane. It functions as a cell surface adhesion molecule by interacting with other ECM-receptor proteins, including ITGB1, laminin family members, and fibronectin 1 (FN1) [13,20]. Many of these genes are associated with clinical outcomes and have been identified as potential diagnostic or prognostic biomarkers in several human cancers [13,16,21,22,23]. However, the association of ITGA3 and other integrin proteins with the prognosis of PCa is little known.

Jiao et al. [16] previously showed that ITGA3 can be used as a prognostic biomarker in PCa patients using bioinformatics analysis. They clarified that ITGA3 expression was greater in PAAD tumors than in control tissues and PCa patients with high ITGA3 levels had significantly poor survival. However, Jiao et al. [16] only analyzed the data from The Cancer Genome Atlas (TCGA) PAAD cohort, without further verification of other datasets. This study aimed to estimate the association of ITGA3 expression with the prognosis in PCa patients using the integrated bioinformatics analysis of multiple microarray datasets. We analyzed the expression profiling of ITGA3 in the TCGA PAAD cohort and Gene Expression Omnibus (GEO) microarray datasets and the association of it with PCa prognosis. Also, a meta-analysis of GEO datasets and the TCGA PAAD cohort was performed to determine the prognostic value of ITGA3 in PCa.

## 2 Materials and methods

### 2.1 Data source

The gene expression RNA-seq in the cohort of GDC TCGA PAAD was extracted from the University of California Santa Cruz (UCSC) Xena (https://xenabrowser.net/datapages/). Also, 14 expression profiling microarray datasets (Table 1) of PCa were selected from the GEO functional genomics data repository (https://www.ncbi.nlm.nih.gov/geo/). Microarray datasets were selected if they met the following inclusion criteria: (1) Homo sapiens; (2) gene expression profile; (3) inclusive of both tumor and adjacent non-tumor tissues, or only tumor samples with survival data. The expression level of ITGA3 (RNA-seq data) was extracted from all datasets. The immunohistochemistry of PCa tumors with ITGA3 protein expression was acquired from the human protein atlas (HPA, https://www.proteinatlas.org/), a Swedish-based program to map all the human proteins in cells, tissues, and organs.

**Table 1:** Characteristics of the 14 datasets included in the analysis

Dataset	Platform	Case number		Country	Survival	PMID
		T	N			
GSE16515	GPL570	36	16	USA	NA	19732725, 27749787, 23936393
GSE22780	GPL570	8	8	USA	NA	/
GSE15471	GPL570	39	39	Romania	NA	19260470, 28881803
GSE71989	GPL570	14	8	USA	NA	27363020
GSE32676	GPL570	25	7	USA	NA	22261810, 25846727
GSE15932	GPL570	16	16	China	NA	/
GSE41372	GPL6244	6	6	Italy	NA	24120476
GSE28735	GPL6244	45	45	USA	Yes	22363658, 23918603
GSE62452	GPL6244	69	61	USA	Yes	27197190
GSE79668	GPL11154	51	/	USA	Yes	27282075
GSE57495	GPL15048	63	/	USA	Yes	26247463
GSE78229	GPL6244	50	/	USA	Yes	27401251
GSE71729	GPL20769	207	134	USA	Yes	26343385
GSE21501	GPL4133	132	/	USA	Yes	20644708, 28380042

T, tumor; N, adjacent non-tumor control.

### 2.2 Functional analysis of the ITGA3 gene

To investigate the functional biological processes and pathways associated with ITGA3, we first identified genes correlated to ITGA3 from the STRING (https://string-db.org/cgi/input.pl). The resulting genes were subsequently submitted to the Database for Annotation, Visualization, and Integrated Discovery (DAVID, version 6.8; https://david.ncifcrf.gov/). Significant categories associated with ITGA3 or ITGA3-related genes were identified if they met the inclusion criteria of hit ≥2 and false discovery rate (FDR) <0.05. Also, pathways related to ITGA3 were identified from the Comparative Toxicogenomics Database (CTD, http://ctdbase.org/).

### 2.3 Statistical analysis

We performed statistical analyses of this study using the SPSS 22.0 software (IBM, Chicago, USA) and RevMan software (version 5.4; Cochrane Collaboration, Oxford, UK). The differences in ITGA3 expression levels between two comparative groups were analyzed using the non-parametric Mann–Whitney *U* test. The association of ITGA3 expression level with the overall survival in PAAD/PCa patients was determined using the univariate and multivariate Cox regression model analysis. We also detected the association of gene expression with PCa prognosis in the gene expression profiling interactive analysis (GEPIA; http://gepia.cancer-pku.cn/index.html). Hazard ratio (HR) and 95% confidence interval (CI) values were calculated. A meta-analysis of the TCGA PAAD cohort and GEO datasets with available survival data was performed to estimate the overall association of ITGA3 expression with the overall survival in PCa patients, with the assessment of heterogeneity across the datasets [24]. For all analyses, the significant criterion was set as *p* < 0.05.

## 3 Results

### 3.1 Characteristics of included data

The characteristics of the included microarrays are shown in Table 1. Ten microarray datasets (GSE16515, GSE22780, GSE15471, GSE71989, GSE32676, GSE15932, GSE41372, GSE28735, GSE62452, and GSE71729) included adjacent non-tumor tissues and tumor samples without overall survival information. Seven microarray datasets (GSE28735, GSE62452, GSE79668, GSE71729, GSE57495, GSE78229, and GSE21501) included tumor samples with clinical data of overall survival (Table 1). The 14 microarray datasets were performed based on six different platforms (GPL570, GPL6244, GPL11154, GPL15048, GPL20769, and GPL4133). Also, patients in 11 microarray datasets were from the United States.

Table 2 shows the clinical data of PAAD patients in the TCGA program. Most tumor tissues were collected from White patients (156/173, 90.17%) with early AJCC pathologic stage (150/174, 86.21%), and head of pancreas (129/177, 72.88%). The TCGA PAAD cohort had a median age of 65 (35–88) years, a median survival length of 438 (0–2741) days, and a median ITGA3 expression level of 5.44 (log2[FPKM + 1]).

**Table 2:** Characteristics of TCGA PCa cohort

Characteristics	Data
Age (year)	65 (35–88)
Gender (male/female)	97/80
Race (White/Black/Asian)	156/6/11
Vital status (death/alive)	92/85
AJCC pathologic T (1/2/3/4)	7/24/141/3
AJCC pathologic N (0/1)	49/123
AJCC pathologic M (0/1)	79/4
AJCC pathologic stage (I/II/III/IV)	21/146/3/4
Prior malignancy (yes/no)	18/159
Prior treatment (yes/no)	1/176
Origin (body/head/tail/other)	15/129/14/19
Survival length (day, median, range)	438 (0–2741)
ITGA3 level (median, range)	5.44 (2.16–7.83)

### 3.2 ITGA3 is upregulated in PAAD tissues

**Figure 1:**
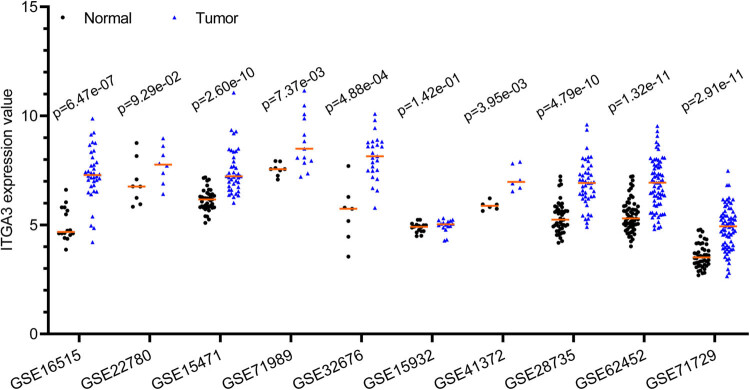
Gene expression profile of ITGA3 in pancreatic cancer tumor samples and adjacent non-tumor tissues. The differences between groups were analyzed using the Mann–Whitney *U* test. The expression levels of ITGA3 are expressed by RMA-normalized expression signal intensity (log2 values; GSE16515, GSE22780, GSE15471, GSE71989, GSE32676, GSE41372, GSE28735, GSE62452, and GSE71729) or variance stabilization normalization (VSN)-normalized signal intensity (log2 values; GSE15932).

**Figure 2:**
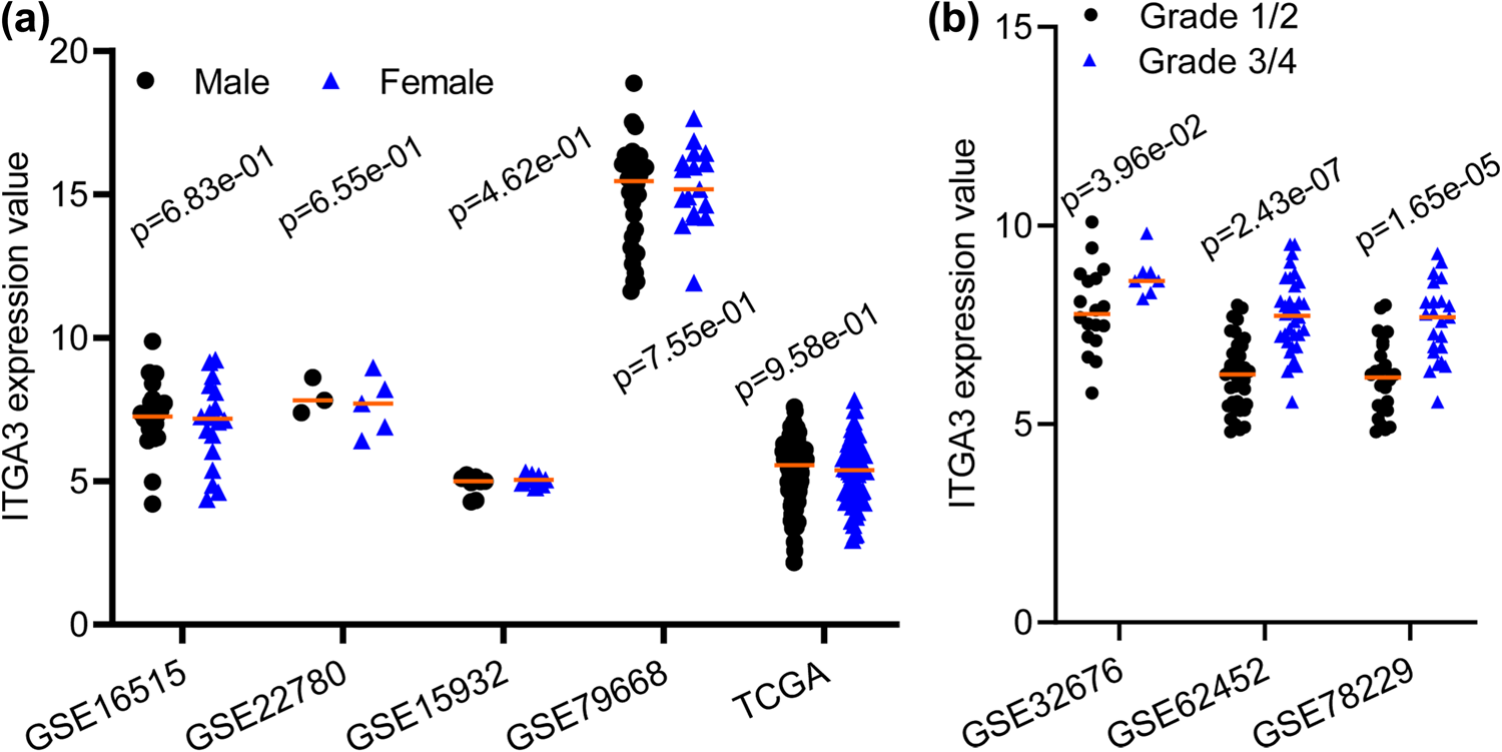
Gene expression profile of ITGA3 in PCa tumor tissues. (a and b) The difference of ITGA3 in PCa tumor tissues collected from male or female patients, or tumor tissues with early or advanced grade, respectively. The differences between groups were analyzed using the Mann–Whitney *U* test. The expression levels of ITGA3 are expressed by RMA-normalized expression signal intensity (log2 values; GSE16515, GSE32676, GSE62452, GSE22780, and GSE78229) log2(Count+1) (GSE79668), VSN-normalized signal intensity (log2 values; GSE15932), or log2 (fragments per kilobase of exon per million fragments mapped, FPKM) (TCGA).

**Figure 3:**
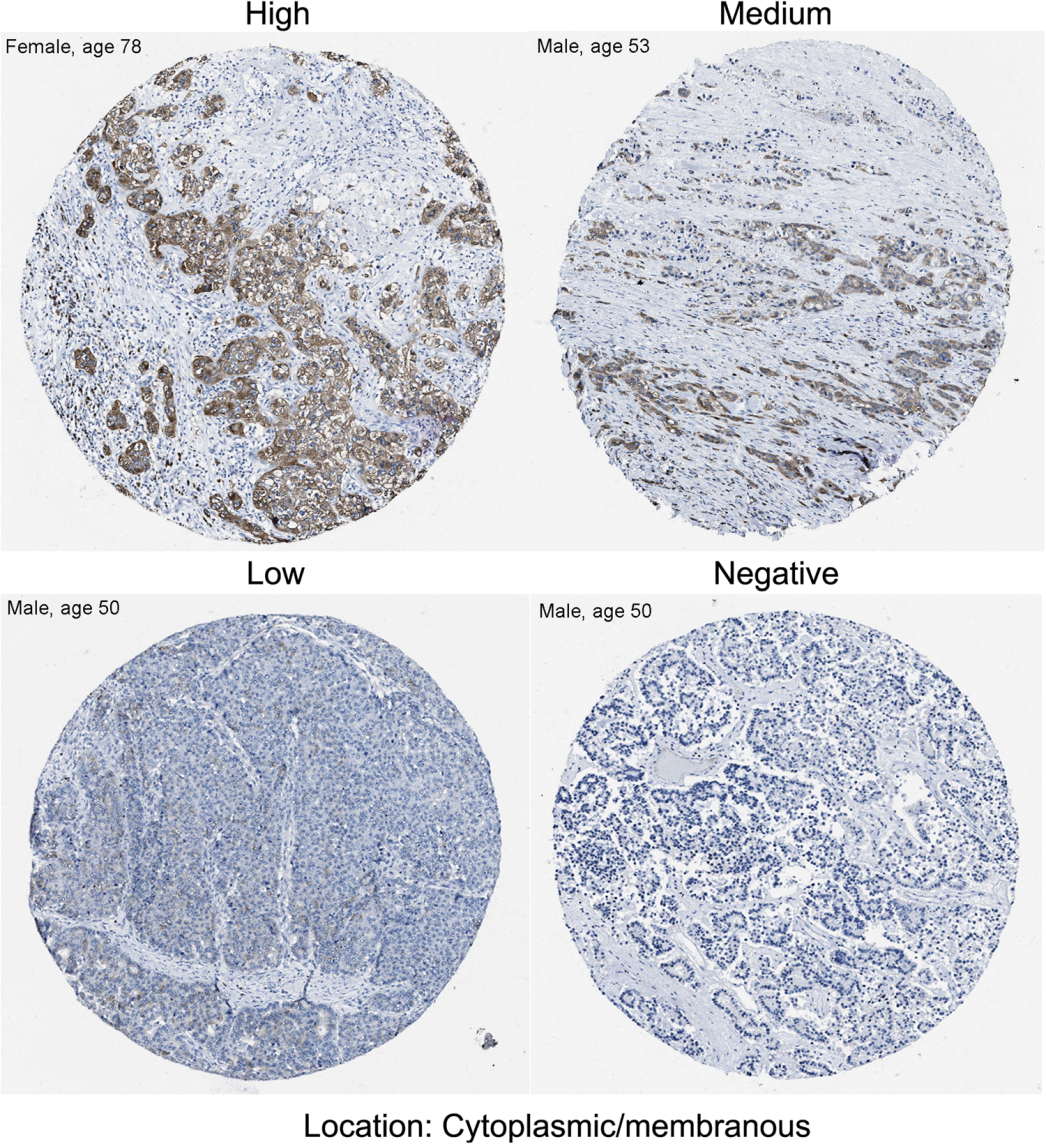
Immunohistochemistry analysis of the ITGA3 protein in the pancreatic cancer tumor tissues. Antibody: CAB018594; Cancer type: Adenocarcinoma. The origin of tissues was Pancreas (T-59000). Staining: High, Medium, Low, and Negative. The representative images of immunohistochemistry of ITGA3 protein were extracted from the human protein atlas (HPA, https://www.proteinatlas.org/).

The results of the Mann–Whitney *U* test of eight microarray datasets showed that PAAD tumor tissues had higher ITGA3 levels than control tissues (*p* < 0.05; Figure 1). There was no difference in ITGA3 levels between female and male patients (Figure 2a). Tumors with advanced grades (3/4) had higher ITGA3 levels than tumors with early-grades (1/2, *p* < 0.05; Figure 2b). The ITGA3 protein levels in PAAD tumor tissues are shown in Figure 3 (staining: high, medium, low, and negative; antibody CAB018594). The expression of this protein varies widely in PCa tumor samples (Figure 3).

### 3.3 Prognostic value of ITGA3 level in PAAD

Cox regression analyses were performed to investigate the potential association of ITGA3 expression in each dataset. Univariate Cox regression model analysis showed that high ITGA3 expression level contributed to a poor prognosis in the TCGA PAAD cohort (HR = 1.464, 95% CI 1.203–1.783, *p* = 1.46 × 10^−4^) and in the PCa patients in datasets GSE79668 (HR = 1.502, 95% CI 1.181–1.911, *p* = 9.12 × 10^−4^), GSE78229 (HR = 1.513, 95% CI 1.122–2.042, *p* = 6.72 × 10^−3^), and GSE62452 (HR = 1.617, 95% CI 1.227–2.131, *p* = 6.50 × 10^−4^; Table 3). However, multivariate Cox regression model analysis showed that ITGA3 expression level was only correlated with PAAD overall survival in TCGA (HR = 1.396, 95% CI 1.135–1.716, *p* = 1.58 × 10^−3^) and in the GSE62452 dataset (HR = 1.450, 95% CI 1.049–2.005, *p* = 2.47 × 10^−2^) after adjusting the confusing factors (age or TNM classifications; Table 3).

**Table 3:** The association of ITGA3 expression with overall survival in patients with PAAD by Cox regression analysis

Datasets	Univariate				Multivariate			
	*β*	HR	95% CI	*P*	*β*	HR	95% CI	*P*
**TCGA**								
Age	0.028	1.028	1.007–1.050	9.59 × 10^−3^	0.027	1.172	1.006–1.049	1.19 × 10^−2^
Gender	−0.164	0.849	0.564–1.279	4.33 × 10^−1^				
Race	0.436	1.276	0.304–5.358	9.33 × 10^−1^				
M	0.006	1.006	0.241–4.206	9.93 × 10^−1^				
N	0.761	2.141	1.274–3.598	4.03 × 10^−3^	0.663	1.941	1.139–3.305	1.47 × 10^−2^
T	0.527	1.694	1.133–2.532	1.02 × 10^−2^	0.159	1.172	0.722–1.904	5.20 × 10^−1^
ITGA3	0.381	1.464	1.203–1.783	1.46 × 10^−4^	0.333	1.396	1.135–1.716	1.58 × 10^−3^
**GSE79668**								
Age	0.025	1.026	0.999–1.053	5.66 × 10^−2^				
Gender	0.213	1.238	0.665–2.304	5.01 × 10^−1^				
M	0.305	1.357	0.184–10.032	7.65 × 10^−1^				
N	0.366	1.442	0.724–2.869	2.97 × 10^−1^				
T	0.300	1.350	0.920–1.982	1.25 × 10^−1^				
ITGA3	0.404	1.502	1.181–1.911	9.12 × 10^−4^				
Diabetes	−0.268	0.765	0.420–1.395	3.82 × 10^−1^				
**GSE78229**								
Grade	0.531	1.701	1.008–2.869	4.66 × 10^−2^	0.237	1.268	0.676–2.377	4.59 × 10^−1^
Stage	−0.451	0.637	0.223–1.824	4.01 × 10^−1^				
ITGA3	0.414	1.513	1.122–2.042	6.72 × 10^−3^	0.307	1.359	0.949–1.946	9.40 × 10^−1^
**GSE62452**								
Grade	0.581	1.787	1.122–2.847	1.45 × 10^−2^	0.250	1.284	0.738–2.234	3.76 × 10^−1^
Stage	0.138	1.148	0.876–1.503	3.17 × 10^−1^				
ITGA3	0.481	1.617	1.227–2.131	6.50 × 10^−4^	0.371	1.450	1.049–2.005	2.47 × 10^−2^
**GSE28735**								
ITGA3	0.213	1.237	0.973–1.573	8.21 × 10^−2^				
**GSE57495**								
Stage	0.438	1.549	0.685–3.502	2.93 × 10^−1^				
ITGA3	0.325	1.385	0.978–1.960	6.70 × 10^−2^				
**GSE21501**								
N	0.614	1.848	1.050–3.253	3.31 × 10^−2^				
T	0.061	1.063	0.622–1.817	8.24 × 10^−1^				
ITGA3	0.229	1.257	0.963–1.641	9.24 × 10^−2^				
**GSE71729**								
ITGA3	0.190	1.209	0.973–1.503	8.73 × 10^−2^				

HR, hazard ratio. CI, confidence interval.

**Figure 4:**
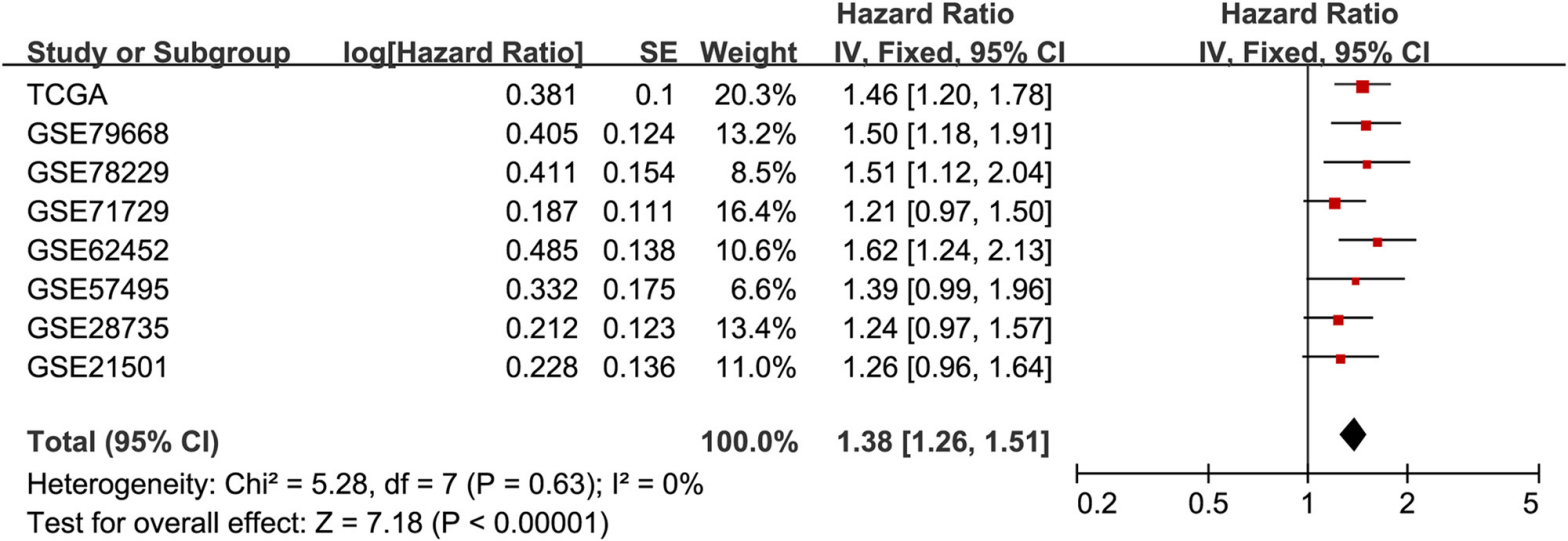
The forest plot indicating the prognostic value of ITGA3 in pancreatic cancer. CI, confidence interval. SE, standard deviation.

A meta-analysis of the TCGA PAAD cohort and seven microarray datasets (GSE28735, GSE62452, GSE79668, GSE71729, GSE57495, GSE78229, and GSE21501) showed that high ITGA3 expression level was a prognostic biomarker of PCa (HR = 1.38, 95% CI 1.26–1.51, *p* < 0.00001; Figure 4). There was no heterogeneity across the datasets (*I* ^*2*^ = 0%).

### 3.4 Prognostic value of ITGA3-related genes in PAAD

**Figure 5:**
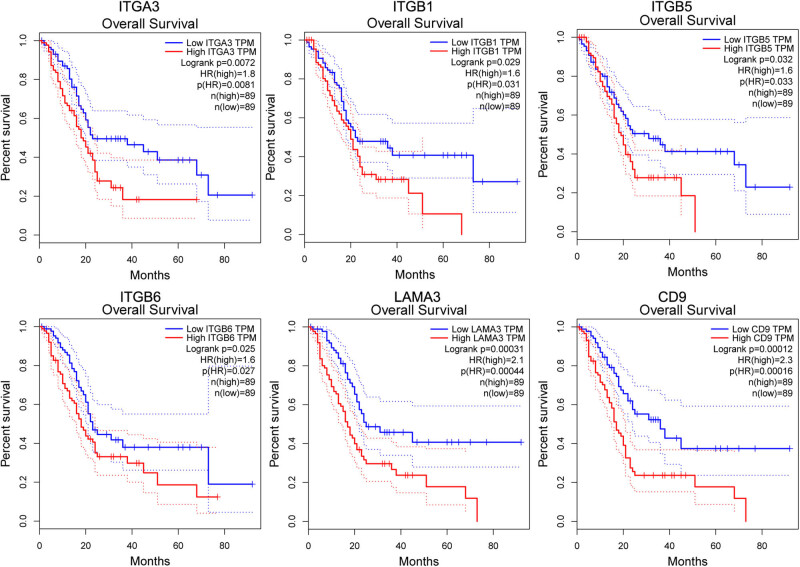
Gene expression profiling interactive analysis (GEPIA) of ITGA3 and related genes. GEPIA performs overall survival analysis using the log-rank test. Dotted line indicates 95% confidence interval.

To further clarify and expand the understanding of the relevance of ITGA3 to PCa prognosis, we first examined the genes related to ITGA3. Ten ITGA3-related genes, including ITGB1, ITGB2, ITGB3, ITGB5, ITGB6, LAMA3, LAMA5, FN1, CD151, and CD9, were identified. GEPIA survival analysis showed that 6 of the 11 genes were associated with overall survival in PAAD significantly (*p* < 0.05; Figure 5). PAAD patients with high expression levels of ITGA3 (HR = 1.8, logrank *p* = 0.0072), ITGB1 (HR = 1.6, logrank *p* = 0.029), ITGB5 (HR = 1.6, logrank *p* = 0.032), ITGB6 (HR = 1.6, logrank *p* = 0.025), LAMA3 (HR = 2.1, logrank *p* = 0.00031), and CD9 (HR = 2.3, logrank *p* = 0.00012) had a worse prognosis compared with patients who had low ITGA3 expression (Figure 5).

### 3.5 ITGA3-related signaling pathways

Functional enrichment analysis and identification in the CTD database showed that the relevance of ITGA3 to seven KEGG pathways, including “hsa04512: ECM-receptor interaction,” “hsa04510: Focal adhesion,” “hsa04810: Regulation of actin cytoskeleton,” and “hsa04151: PI3K-Akt signaling pathway” (Table 4).

**Table 4:** Functional enrichment analysis for the pathways related to ITGA3

KEGG pathways	Genes	*P* Value	FDR
hsa04512: ECM-receptor interaction	ITGB1, ITGB5, ITGA3, ITGB3, LAMA3, FN1, ITGB6	9.44 × 10^−11^	2.26 × 10^−9^
hsa04510: Focal adhesion	ITGB1, ITGB5, ITGA3, ITGB3, LAMA3, FN1, ITGB6	1.79 × 10^−8^	1.61 × 10^−7^
hsa04810: Regulation of actin cytoskeleton	ITGB1, ITGB5, ITGA3, ITGB3, ITGB2, FN1, ITGB6	2.01 × 10^−8^	1.61 × 10^−7^
hsa04151: PI3K-Akt signaling pathway	ITGB1, ITGB5, ITGA3, ITGB3, LAMA3, FN1, ITGB6	3.92 × 10^−7^	2.35 × 10^−6^
hsa05412: Arrhythmogenic right ventricular cardiomyopathy (ARVC)	ITGB1, ITGB5, ITGA3, ITGB3, ITGB6	5.59 × 10^−7^	2.68 × 10^−6^
hsa05410: Hypertrophic cardiomyopathy (HCM)	ITGB1, ITGB5, ITGA3, ITGB3, ITGB6	1.03 × 10^−6^	4.14 × 10^−6^
hsa05414: Dilated cardiomyopathy	ITGB1, ITGB5, ITGA3, ITGB3, ITGB6	1.40 × 10^−6^	4.79 × 10^−6^

FDR, false discovery rate.

## 4 Discussion

The association of ITGA3 expression with the prognosis in PCa patients has been identified in a previous study by Jiao et al. [16]. Similar to the results of the above article, we verified that this gene was highly expressed in PCa tumors as compared with the normal controls. Notably, we showed that ITGA3 expression was elevated in PCa tumor tissues compared with controls in eight microarray datasets and the TCGA PAAD cohort. Also, a meta-analysis of seven microarray datasets and the TCGA PAAD cohort verified the remarkable overall association of ITGA3 with PCa prognosis (HR = 1.38, 95% CI 1.26–1.51, *p* < 0.00001), indicting ITGA3 was a potential prognostic biomarker in PCa.

ITGA3 functions by interacting with other cell surface adhesion molecules and ECM-receptor proteins, including ITGB1 and FN1 [13,18,20]. These factors are associated with cancer cell proliferation, migration, invasion, and cancer metastasis [25,26,27,28]. Koshizuka et al. [27] showed that ITGA3 elevation was related to worse overall survival in patients with head and neck squamous cell carcinoma. Also, Tang et al. [28] showed that high ITGA3 expression correlated with poor overall survival in patients with nasopharyngeal carcinoma. Idichi et al. [29] also found that high ITGA3 expression was associated with poor prognosis, recurrence, and increased lymph node metastasis in PCa patients. Our present study showed that the expression level of ITGA3 in PCa tumor tissues was greater than that in the adjacent non-tumor tissues. The meta-analysis of seven microarray datasets and the TCGA PAAD cohort showed that high ITGA3 expression related to a poor prognosis in PCa (HR = 1.38, 95% CI 1.26–1.51, *p* < 0.00001). The evidence indicates that ITGA3 is a potential prognostic marker in PCa.

ITGA3 is an ECM receptor that functions as a cell surface adhesion molecule [13,20]. It regulates a variety of biological processes via signaling pathways, like the phosphoinositide 3-kinase (PI3K)-Akt signaling pathway [30]. Koshizuka et al. [27] showed that ITGA3 inhibition significantly inhibited the migration and invasion of cancer cells. Zhang et al. [30] showed that ITGA3 activates the proliferation, invasion, and migration by activating the PI3K-Akt signaling pathway in breast cancer cells. The knockdown of ITGA3 inactivated the PI3K-Akt axis signaling pathway. Wang et al. [26] confirmed that the inhibition of ITGA3 significantly suppressed the phosphorylation levels of focal adhesion kinase (FAK), PI3K, and Akt in bladder cancer cells. Idichi et al. [29] indicated that the phosphorylation levels of FAK, Akt, and ERK1/2 were suppressed by miR-124-3p through inhibiting ITGA3. Also, the inhibition of SRPX2 suppressed the PI3K/AKT/mTOR signaling and the proliferation, invasion, migration, and chemoresistance of PC cells [31]. These results showed that ITGA3 inhibition could be a potential therapeutic target for PCa, inhibition of ITGA3 expression can suppress cell proliferation, migration, and invasion. Moreover, our study showed that ITGA3 was enriched in the pathways including ECM-receptor interaction, Focal adhesion, and PI3K-Akt signaling pathway. These results showed that ITGA3 mediates the PCa cell proliferation and tumor progression by multiple signaling pathways, including the PI3K-Akt signaling pathway.

## 5 Conclusion

In summary, this study showed that ITGA3 expression was greater in PCa tumor tissues than in adjacent non-tumor tissues. The Cox regression analysis showed that ITGA3 elevation contributed to worse overall survivals of PCa patients in most included datasets. However, the meta-analysis of the TCGA PAAD cohort and seven microarray datasets (GSE28735, GSE62452, GSE79668, GSE71729, GSE57495, GSE78229, and GSE21501) showed that ITGA3 expression related to a poor prognosis in PCa. We found that the relevance of ITGA3 with PCa prognosis was mediated by the PI3K-Akt signaling pathway. However, more preclinical experiments and clinical trials need to be completed to estimate the possibility of using ITGA3 as a clinical prognostic biomarker for PCa.
